# An observational study of the association between microalbuminuria and increased N-terminal pro-B-type natriuretic peptide in patients with subarachnoid hemorrhage

**DOI:** 10.1186/s40560-015-0108-1

**Published:** 2015-10-14

**Authors:** Yoshiaki Terao, Makito Oji, Tomomi Toyoda, Haruka Inoue, Makoto Fukusaki, Tetsuya Hara

**Affiliations:** Department of Anesthesia, Nagasaki Rosai Hospital, 2-12-5 Setogoe, Sasebo, 857-0134 Japan; Department of Anesthesiology, Nagasaki University School of Medicine, 1-7-1 Sakamoto, Nagasaki, 852-8501 Japan

**Keywords:** Microalbuminuria, Subarachnoid hemorrhage, B-type natriuretic peptide, Neurological outcome, N-terminal pro-B-type natriuretic peptide

## Abstract

**Background:**

The urinary albumin/creatinine ratio (ACR) is a significant neurologic prognostic predictor in patients with aneurysmal subarachnoid hemorrhage (SAH). B-type natriuretic peptide (BNP) plays an important role in body fluid regulation in patients with SAH. The present study was performed to determine whether ACR was independent predictor for unfavorable neurological outcome and ACR was associated with increased N-terminal pro-BNP (NT-pro-BNP) after SAH.

**Methods:**

We studied 61 patients undergoing surgery who were admitted within 48 h after aneurysmal SAH onset between July 2008 and June 2010. Hunt and Hess grade and Fisher grade were recorded at admission. The Glasgow Coma Scale (GCS) score was calculated at admission and daily for seven postoperative days. Arterial blood was sampled at admission and for seven postoperative days to determine the PaO_2_/F_I_O_2_ ratio, C-reactive protein level, troponin I level, and NT-pro-BNP level. Urine was sampled at admission and daily for seven postoperative days to determine ACR and vanillylmandelic acid/creatinine ratio (VMACR). Neurological outcomes were assessed at hospital discharge by using the Glasgow Outcome Scale. Receiver operating characteristic curves were constructed for the predictive variables of unfavorable neurological outcomes, and the area under the curve (AUC) was determined. Multivariate logistic regression analyses were performed for the significant predictors of unfavorable neurological outcomes after SAH. Associations with NT-pro-BNP were evaluated by using the Spearman rank correlation test.

**Results:**

Of the 61 patients, 24 had unfavorable outcomes. The prevalence rate of microalbuminuria was 85 % (52/61). The highest NT-pro-BNP levels were above the normal range in 57 of 61 patients (93 %).

According to the AUC, the Hunt and Hess grade, GCS score, the highest ACR, and highest VMACR were significant predictors of neurological outcome. Multivariate logistic regression analyses showed that the highest ACR and Hunt and Hess grade are independent prognostic predictors of unfavorable neurological outcomes. The highest NT-pro-BNP significantly correlated with the highest troponin I, highest ACR, and VMACR on admission.

**Conclusions:**

The highest ACR is an independent prognostic predictor of unfavorable neurological outcomes after SAH. Moreover, plasma NT-pro-BNP elevation may be associated with the development of microalbuminuria.

## Background

Patients with aneurysmal subarachnoid hemorrhage (SAH) [1], intracerebral hemorrhage [2], and cerebral infarction [3] show a high prevalence of microalbuminuria. The microalbuminuria, measured by urinary albumin/creatinine ratio (ACR), is a significant prognostic predictor in patients with SAH and intracerebral hemorrhage [1, 2]. However, our previous pilot study did not determine whether ACR was an independent prognostic predictor in patients with SAH [1]. Furthermore, the underlying mechanism of the association between hemorrhagic stroke and increased ACR was unclear.

Myocyte stretch leads to the release of a prohormone, which is subsequently split into B-type natriuretic peptide (BNP) and an N-terminal pro-BNP (NT-pro-BNP), a biologically inactive molecule. The increase in BNP levels plays an important role in the regulation of body fluid in critically ill patients, including patients with SAH [4]. One study showed that the BNP levels increased gradually over 2 days and returned to normal within a week after SAH [5]. Plasma BNP and NT-pro-BNP might be associated with cardiac dysfunction and myocardial necrosis in the acute phase of SAH [5, 6]. Moreover, microalbuminuria is associated with cardiovascular disease in patients with diabetes mellitus [7] and in the general population [8]. Although an increased BNP level correlated with microalbuminuria in patients with diabetes mellitus [9], it is unknown whether this relationship applies to patients with SAH.

The present prospective observational study was performed to determine whether ACR was independent predictor for unfavorable neurological outcome and the development of ACR was associated with increased NT-pro-BNP levels after SAH.

## Methods

### Patients

The study protocol was approved by the Institutional Research and Ethics Committee. In accordance with the Helsinki Declaration, informed consent was obtained from all patients or the patients’ next of kin. Between July 2008 and June 2010, we prospectively studied 61 consecutive patients who were admitted to the Nagasaki Rosai Hospital within 48 h after SAH onset. The exclusion criteria included the presence of renal dysfunction (serum creatinine level >1.2 mg/dL), which affects ACR values regardless of changes in glomerular permeability [10]; no treatment administered for a ruptured aneurysm, because of poor neurological condition (Hunt and Hess grade V) or either because the aneurysm was not verified by cerebral angiography; and continuous postoperative barbiturate coma, which did not allow the determination of a Glasgow Coma Scale (GCS) score. An initial computed tomography (CT) scan of the head was performed on admission. The diagnosis was established on the basis of the CT scan findings or by observing xanthochromia of the cerebrospinal fluid when results of the CT scan were negative. Thereafter, cerebral four-vessel angiography and/or three-dimensional CT were performed. Surgical clipping of the aneurysm or endovascular surgery was performed at the earliest possible time, as determined by a team of neurosurgeons experienced in both treatment modalities. Patients underwent surgery for ruptured aneurysms within 72 h after stroke. Anesthesia was induced with propofol and fentanyl and maintained with sevoflurane, fentanyl, and vecuronium bromide.

### Clinical management

Postoperatively, all patients received conventional brain-oriented intensive care therapy according to clinical requirements. Normovolemia was maintained through the systemic administration of an intravenous physiological electrolyte solution at 1000–2000 mL/day and a colloid solution as needed. Symptomatic hypovolemia was corrected by the administration of additional fluids, followed by a continuous infusion of dopamine, as needed—to maintain a systolic arterial blood pressure of >100 mmHg. The systolic arterial blood pressure was maintained either at <160 mmHg in normotensive patients or at <180 mmHg in hypertensive patients via continuous infusion of nicardipine as needed. Caution was taken to avoid hyperthermia (>38.5 °C) and hyperglycemia (>150 mg/dL) through the administration of diclofenac sodium and insulin as needed. In mechanically ventilated patients, the arterial carbon dioxide partial pressure was maintained at 35–40 mmHg, and the peripheral oxygen saturation was maintained at >97 %—under sedation with midazolam if required. Oral and enteral nutrition was initiated as soon as possible. A CT scan was performed in clinically deteriorating patients to identify secondary complications such as hydrocephalus or ischemia. Hypertensive hypervolemic hemodilution (triple-H) therapy was not used for prophylaxis. Hydrocephalus was treated with ventriculostomy. Delayed cerebral ischemia was suspected in the event of neurological deterioration (two-point decrease in the GCS score and/or focal neurological deficits) and confirmed by cerebral angiography [11]. Hypertensive therapy was reinforced with dobutamine in each vasospasm episode. The target systolic blood pressure was 160–180 mmHg. When technically possible, angioplasty was performed in patients who failed to respond to therapy.

### Data collection

The following clinical data was collected: age, sex, previous medical history of hypertension, ischemic heart disease, heart failure and diabetes mellitus, size and location of the aneurysm, and systolic blood pressure. The location of the ruptured aneurysms was classified according to their presence in the anterior or posterior circulation [12]. The aneurysm size was categorized as large (>12 mm) or other [12]. The neurological condition on admission was scored according to the Hunt and Hess classification [13]. The amount of blood observed on the initial CT scan was graded according to the Fisher classification [14]. The GCS score [15] and systemic inflammatory response syndrome (SIRS) score [16] were calculated at admission and daily for seven postoperative days. All physiological variables were recorded as the most abnormal value obtained in a day, except those recorded on admission. Arterial blood was sampled at admission and for seven postoperative days to determine the PaO_2_/F_I_O_2_ ratio, C-reactive protein (CRP) level, troponin I level, and NT-pro-BNP level. Serum troponine I levels were assayed by using chemiluminescence immunoassay (Architect Troponin-I, Abbott Laboratories, Abbott Park, IL) with an applied threshold of 0.30 ng/mL for the diagnosis of myocardial necrosis (a lower limit of detection of 0.01 ng/mL; coefficient variation, 10 %). Serum NT-pro-BNP levels were assayed by performing electrochemiluminescence immunoassay (Roche Diagnostics, Indianapolis, IN) with an applied threshold of 125 pg/mL (a lower limit of detection of 5 pg/mL; coefficient variation, 10 %). The ACR and vanillylmandelic acid/creatinine ratio (VMACR) measurements were determined from urine samples collected at admission and daily for seven postoperative days. We assayed urine vanillylmandelic acid by using high-performance liquid chromatography with a lower limit of detection of 0.05 mg/dL and a coefficient variation of 2.9 %. VMACR values of <5 mg/g were considered normal. We quantitatively assessed urine albumin by using an immunonephelometric method (N-antiserum albumin, Dade Behring, Liederbach, Germany), and we quantitatively measured urine creatinine by performing an enzymatic colorimetric test. The sensitivity limit for urine albumin was 2.3 mg/L; for statistical analyses, values below this limit were considered to be 0 mg/L. Both inter-assay and intra-assay coefficient variations were within 5 %. Generally, clinically relevant proteinuria is defined as an ACR of ≥300 mg/g and microalbuminuria, as an ACR of 30–299 mg/g; values <30 mg/g are considered normal. A medical chart review was conducted to determine the duration of mechanical ventilation, intensive care unit stay, and hospital stay. The neurosurgeons used the Glasgow Outcome Scale (GOS) [17] to assess the neurological outcomes at hospital discharge. Neurological outcomes were stratified as unfavorable (GOS score of 1–3: death, persistent vegetative state, and severe disability) or favorable (GOS score of 4 or 5: moderate disability and good recovery) [1, 18]. In the present study, the intensive care unit care providers (neurosurgeons and nursing staff) were blinded to the ACR and NT-pro-BNP values. The authors were the patients’ anesthesiologists who were responsible only for blood and urine sampling and the aforementioned assays.

### Statistical analysis

Data distributions for the quantitative variables were expressed as median (interquartile range). Intergroup comparisons were performed using the Mann-Whitney *U* test. Dichotomous variables were analyzed with the Fisher exact probability test or the chi-squared test. To assess the predictive value for neurological outcomes, receiver operating characteristic (ROC) curves were constructed for each variable and the area under the ROC curve (AUC) was determined. Predictive variables were considered significant predictors when the AUC was >0.8. The threshold value, sensitivity, specificity, and likelihood ratio of the ACR were compared with those of some significant predictors of unfavorable neurological outcomes. Multivariate logistic regression analyses were performed, including significant predictors and dichotomized by its threshold value base on ROC analyses with stepwise elimination of valuables not contributing to the model (*p* < 0.1). Associations between the ACR or the NT-pro-BNP level and other variables were evaluated using the Spearman rank correlation test.

The sample size of 22 patients with SAH per group (standard deviation, 64 mg/g) was determined on the basis of our previous study [1], which reflected a power of 90 % to detect a 100 % difference in the highest ACR between the two neurological outcomes at a significance level of 5 %.

## Results

**Fig. 1:**
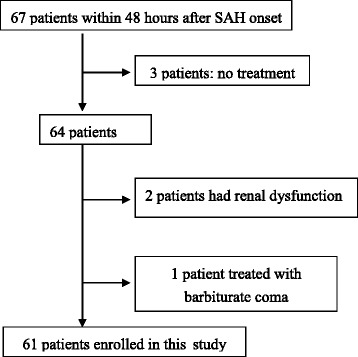
The flowchart. Flowchart of patients into study. *SAH* subarachnoid hemorrhage

During the study period, 67 consecutive patients were admitted to the ICU within 48 h after aneurysmal SAH. Of these, we excluded 3 patients who did not receive any treatment for the ruptured aneurysm due to poor neurological condition, 2 patients due to renal dysfunction, and 1 patients who was treated with barbiturate coma. A flowchart documenting patient’s entry into the study is shown in Fig. 1.

The prevalence rate of microalbuminuria (>30 mg/g) was 85 % (52/61). Of the 61 patients enrolled in the study, 8 (13.1 %) died (GOS score of 1). Of the 53 patients who were discharged, 16 (26 %) had severe disability (GOS score of 3), 11 (19 %) had moderate disability (GOS score of 4), and 26 (43 %) showed good recovery (GOS score of 5). Thus, 24 of the 61 study patients (39 %) had unfavorable outcomes. The highest NT-pro-BNP levels were above the normal range (125 pg/mL) in 57 of 61 patients (93 %).

Patient characteristics are shown in Table 1. The age, Fisher grade, and Hunt and Hess grade were higher in the patients with unfavorable outcomes than in those with favorable outcomes. Table 2 shows the relationship between neurological outcomes and selected laboratory variables. The ACR on admission, highest ACR, VMACR on admission, highest VMACR, highest CRP level, highest troponin I, NT-pro-BNP level on admission, and highest NT-pro-BNP level were higher in patients with unfavorable neurological outcomes than in those with favorable neurological outcomes. The GCS score on admission and the lowest GCS score were lower in the patients with unfavorable outcomes than in those with favorable outcomes. The highest SIRS score, the prevalence delayed cerebral ischemia, and mechanical ventilation duration were higher in the patients with unfavorable outcomes than in those with favorable outcomes.

**Table 1:** Relationship between neurological outcome and patient characteristics

Variables	Favorable outcome	Unfavorable outcome	*p*
Number	37	24	
Age	63 (54, 73)	74 (59, 83)	0.03
Female (n)	26 (70.3)	20 (83.3)	0.25
Height (cm)	155 (150, 163)	150 (146, 159)	0.09
Weight (kg)	55 (47, 62)	50 (44, 60)	0.15
Body mass index (cm^2^)	21.9 (21.0, 24.9)	20.8 (19.7, 24.6)	0.17
Hypertension (n)	14 (37.8)	13 (54.2)	0.21
Ischemic heart disease (n)	1 (2.7)	0 (0)	1.0
Heart failure (n)	0 (0)	1 (4.2)	1.0
Diabetes (n)	1 (2.7)	0 (0)	1.0
Fisher grade	3 (3, 3)	3 (3, 4)	<0.01
Hunt and Hess grade	2 (2, 3)	4 (3, 5)	<0.01
Large aneurysmal size (n)	3 (8.1)	1 (4.2)	0.65
Posterior aneurysmal location (n)	6 (16.2)	5 (20.8)	0.65
Admission SBP (mmHg)	158 (140, 169)	149 (122, 174)	0.21
Endovascular surgery (n)	7 (18.9)	9 (37.5)	0.67

Values are median (interquartile range) or number (percentage). Favorable outcome (Glasgow Outcome Scale: moderate disability and good recovery) and unfavorable outcome (Glasgow Outcome Scale: death, persistent vegetative state, and severe disability

*SBP* systolic blood pressure

**Table 2:** Relationship between neurological outcome and the selected variables

Variables	Favorable outcome	Unfavorable outcome	*p*
ACR (mg/g) on admission	64.7 (22.5, 119.3)	222.8 (58.1, 556.6)	<0.01
Highest ACR (mg/g)	113.3 (32.7, 220.7)	497.5 (239.6, 1091.7)	<0.01
VMACR (mg/g) on admission	5.2 (3.5, 7.8)	9.1 (5.7, 12.2)	<0.01
Highest VMACR (mg/g)	5.6 (4.1, 8.1)	10.9 (8.2, 15.3)	<0.01
Highest C-reactive protein (mg/dL)	3.4 (1.7, 6.8)	6.9 (1.8, 13.8)	0.02
Highest serum creatinine (mg/g)	0.7 (0.6, 0.8)	0.7 (0.5, 0.9)	0.74
Lowest P_a_O_2_/F_I_O_2_ ratio	312 (287, 380)	325 (192, 369)	0.24
Highest troponin I (ng/mL)	0.0 (0.0, 0.0)	0.1 (0.0, 0.6)	0.03
NT-pro- BNP on admission (pg/mL)	220 (117, 508)	542 (172, 1732)	0.03
Highest NT-pro-BNP (pg/mL)	703 (238, 1216)	1509 (759, 3438)	<0.01
GCS score on admission	15 (13, 15)	10 (7, 14)	<0.01
Lowest GCS score	13 (13, 14)	8 (4, 10)	<0.01
Highest SIRS score	2 (1, 2)	2 (2, 3)	<0.01
Hydrocephalus (n)	13 (8.1)	12 (50.0)	0.25
Delayed cerebral ischemia (n)	9 (24.3)	12 (50.0)	0.04
Lung edema (n)	7 (18.9)	9 (37.5)	0.11
Ventilation days	0 (0, 1)	5 (1, 15)	<0.01
Intensive care unit days	14 (12,16)	16 (12, 21)	0.10
Hospital days	40 (24, 53)	53 (25, 74)	0.29

Values are median (interquartile range) or number. Favorable outcome (Glasgow Outcome Scale: moderate disability and good recovery) and unfavorable outcome (Glasgow Outcome Scale: death, persistent vegetative state, and severe disability)

*ACR* urinary microalbumin/creatinine ratio, *VMACR* urinary vanillylmandelic acid/creatinine ratio, *NT-pro-BNP* N-terminal pro-Brain natriuremic peptide, *GCS* Glasgow Coma Scale, *SIRS* systemic inflammatory response syndrome

The AUC was >0.8 for the Hunt and Hess grade, GCS score on admission, lowest GCS score, highest ACR, and highest VMACR (Table 3). Table 4 shows a comparison of the predictive characteristics of the significant predictors of neurological outcomes in patients with SAH. Table 5 summarizes the results of multivariate logistic regression analyses of these five significant predictors based on ROC. The results shows the ACR (>280 mg/g) and Hunt-Hess grade (>3) are independently associated with unfavorable neurological outcome.

**Table 3:** The area under the receiver characteristics curves constructed for each of the predictive variables of neurological outcome at hospital discharge

Variables	Area (95 % CI)
Hunt and Hess grade	0.81 (0.69–0.93)^a^
Fisher grade	0.70 (0.57–0.83)
Glasgow Coma Scale score on admission	0.83 (0.72–0.94)^a^
Lowest Glasgow Coma Scale	0.87 (0.77–0.97)^a^
Delayed cerebral ischemia	0.63 (0.48–0.78)
Highest troponin I	0.67 (0.52–0.82)
Highest systemic inflammatory response syndrome score	0.70 (0.57–0.83)
Urinary albumin/creatinine ratio on admisssion	0.75 (0.62–0.88)
Highest urinary albumin/creatinine ratio	0.84 (0.73–0.95)^a^
Urinary vanillylmandelic acid/creatinine ratio on admission	0.75 (0.63–0.87)
Highest urinary vanillylmandelic acid/creatinine ratio	0.82 (0.71–0.93)^a^
N-terminal pro brain natriuremic peptide on admission	0.67 (0.52–0.82)
Highest N-terminal pro brain natriuremic peptide	0.73 (0.60–0.86)

*Area* area under the receiver characteristics curve, *CI* confidence interval

^a^Each predictive variable was defined as a significant predictor when areas of the variables were more than 0.8

**Table 4:** Comparison of significant predictor’s characteristics that predict unfavorable neurologic outcome at hospital discharge after subarachnoid hemorrhage

Variables	Threshold value	Sensitivity (95 % CI) (%)	Specificity (95 % CI) (%)	Likelihood ratio (95 % CI)
Hunt-Hess grade	>3	58 (52–64)	92 (89–95)	7.2 (4.0–13.0)
Highest ACR (mg/g)	>280	63 (56–69)	97 (95–99)	23.1 (12.7–42.1)
GCS score on admission	<10	42 (36–48)	97 (95–99)	15.4 (9.5–25.0)
Lowest GCS score	<9	42 (33–67)	97 (95–99)	15.4 (9.5–25.0)
Highest VMACR (mg/g)	>14	38 (32–44)	97 (95–99)	13.9 (8.7–22.3)

Unfavorable outcome (Glasgow Outcome Scale: death, persistent vegetative state, and severe disability)

*CI* confidence interval, *ACR* urinary albumin/creatinine ratio, *GCS* Glasgow Coma Scale, *VMACR* urinary vanillylmandelic acid/creatinine ratio

**Table 5:** Multivariate logistic regression analyses of predictive factors for unfavorable neurologic outcome at hospital discharge after comparison of the predictive characteristics of the significant predictor

Variables	Threshold value	Odds ratio (95 % CI)	*p*
Hunt-Hess grade	>3	19.2 (1.7–217.2)	0.02
Highest ACR (mg/g)	>280	30.7 (3.3–284.4)	0.01
GCS score on admission	<10	1.1 (0.1–27.1)	0.96
Lowest GCS score	<9	1.3 (0.1–14.5)	0.86
Highest VMACR (mg/g)	>14	1.5 (0.1–19.0)	0.74

Unfavorable outcome (Glasgow Outcome Scale: death, persistent vegetative state, and severe disability)

*CI* confidence interval, *ACR* urinary albumin/creatinine ratio, *GCS* Glasgow Coma Scale, *VMACR* urinary vanillylmandelic acid/creatinine ratio

The highest ACR was significantly correlated with the age (*r* = 0.42), Hunt and Hess grade (*r* = 0.49), GCS score on admission (*r* = −0.49), lowest GCS score (*r* = −0.56), highest CRP level (*r* = 0.41), highest SIRS (*r* = 0.51), highest troponin I level (*r* = 0.47), ACR on admission (*r* = 0.77), VMACR on admission (*r* = 0.53), highest VMACR (*r* = 0.51), NT-pro-BNP level on admission (*r* = 0.39), highest NT-pro-BNP level (*r* = 0.56), mechanical ventilation duration (*r* = 0.53), and GOS score (*r* = 0.62). The highest NT-pro-BNP level was significantly correlated with the highest troponin I level (*r* = 0.62), NT-pro-BNP level on admission (*r* = 0.63), highest ACR (*r* = 0.56), and VMACR on admission (*r* = 0.53).

## Discussion

The results of the present study suggest that plasma NT-pro-BNP elevation may associated with the development of microalbuminuria as well as cardiac injury and sympathetic nervous system activation after SAH, because the highest NT-pro-BNP level was significantly correlated with the highest ACR, highest troponin I level, and VMACR on admission. Moreover, our findings show that the ACR and the Hunt and Hess grade are independent prognostic predictors of unfavorable neurological outcomes after SAH.

The BNP concentrations increased in critically ill patients [19]. The highest BNP concentrations were observed in patients who underwent cardiac surgery procedures and in patients with SAH. Elevated BNP levels are associated with myocardial necrosis, pulmonary edema, and systolic and diastolic dysfunction of the left ventricle after SAH [6]. On the other hand, an echocardiographic study showed that plasma BNP was associated with myocardial necrosis but was unrelated with systolic and diastolic cardiac function [4]. The present study showed that the plasma NT-pro-BNP levels were associated with myocardial necrosis evaluated by troponin I levels. However, it was not unclear whether the plasma NT-pro-BNP levels were associated with systolic and diastolic cardiac dysfunction, because we did not conduct the echocardiographic analysis. The heart is the primary source of elevated BNP levels after SAH [6]. However, the correlation between the BNP elevation and illness severity or mortality is controversial.

The present study showed that plasma NT-pro-BNP levels were associated with VMACR levels. The increase in BNP levels after SAH owing to increased cardiac production may have been triggered by stress-induced noradrenaline release [20]. Moreover, hypothalamus lesions caused by SAH may induce expanded cardiac BNP production. The overactivity of the sympathetic nervous system is a common phenomenon that links the major cardiac pathologies observed in neurological disorders. The stimulation of the hypothalamus can lead to autonomic cardiovascular disturbances, known as the “the brain-heart connection” [21].

Moreover, the present study showed that plasma NT-pro-BNP levels were related to ACR. Yano et al. showed that the increased plasma BNP levels were correlated with the urinary albumin excretion rate in normotensive patients with diabetes mellitus [9]; however, contrasting results were reported by Asakawa et al. [22]. Although the relationship between BNP and microalbuminuria levels remains unclear in patients with diabetes mellitus, the infusion of atrial natriuretic peptide increases the urinary excretion of albumin in patients with diabetes mellitus [23]. Atrial natriuretic peptide is known to exert a vasodilatory effect on the afferent arteries and a vasoconstrictor effect on the efferent arteries of the glomeruli, thereby increasing the glomerular hydraulic pressure. Given that BNP and atrial natriuretic peptide bind to the same receptor and have the same biological activity, the elevated levels of BNP cause an increase in the glomerular hydraulic pressure and ultimately induce albumin excretion in patients with diabetes mellitus [9].

Age, admission neurological grade, history of hypertension, systolic blood pressure at admission, ruptured aneurysm location and size, blood clot thickness on CT scans, and angiographic vasospasm at admission are recognized as neurological prognostic factors in multicenter clinical study with a large number of aneurysmal SAH patients [12]. Delayed cerebral ischemia, intraventricular hemorrhage, rebleeding, medical complication including fever, anemia, hypertension, respiratory failure and cardiac complication, and the SIRS score and Acute Physiology and Chronic Health Evaluation 2 score were associated with neurological outcome in SAH patients [16, 24–27]. The present results show that among these prescribed predictors, the highest ACR and the Hunt-Hess grade are the independent predictors of neurological outcome. However, Hunt-Hess grade is a subject assessment. Measurement of the ACR during first 8 days may provide objective and reliable information for the neurological outcome in the SAH patients.

### Limitations

Although we measured ACR in a spot urine sample, Gansevoort et al. [28] showed that the diagnostic performance of measuring ACR in a spot urine sample in predicting microalbuminuria in a subsequent 24-h urine collections was clinically satisfactory.

This study showed the significant and moderate correlations between the ACR and the plasma NT-pro-BNP. It is unclear whether plasma NT-pro-BNP elevation contributes to the development of microalbuminuria in this study results. Further studies are necessary to clarify the exact relationship between plasma NT-pro-BNP elevation and the development of microalbuminuria in the SAH patients.

## Conclusions

The highest ACR is an independent prognostic predictor of unfavorable neurological outcomes. Although the mechanism of the development of microalbuminuria in unfavorable neurological patients after SAH is unknown at present, this study provided the information that the plasma NT-pro-BNP elevation might be associated with the development of microalbuminuria after SAH. Further studies are necessary to better understand the mechanism of the development of microalbuminuria in the SAH patients.

## Abbreviations

ACR: urinary albumin/creatinine ratio
AUC: area under the curve
BNP: B-type natriuretic peptide
CRP: C-reactive protein
CT: computed tomography
GCS: Glasgow Coma Scale
GOS: Glasgow Outcome Scale
NT-pro-BNP: N-terminal pro-B-type natriuretic peptide
ROC: receiver operating characteristic
SAH: subarachnoid hemorrhage
SIRS: systemic inflammatory response syndrome
VMACR: vanillylmandelic acid/creatinine ratio
